# Evaluation of clinical knowledge and perceptions about the development of thyroid cancer—An observational study of healthcare undergraduates in Saudi Arabia

**DOI:** 10.3389/fpubh.2022.912424

**Published:** 2022-08-15

**Authors:** Wajid Syed, Osama A. Samarkandi, Ahmed Alsadoun, Mohammad K. Al Harbi, Mahmood Basil A. Al-Rawi

**Affiliations:** ^1^Department of Clinical Pharmacy, College of Pharmacy, King Saud University, Riyadh, Saudi Arabia; ^2^Nursing Informatics Vice Dean for Academic Affairs, Prince Sultan College for Emergency Medical Services, King Saud University, Riyadh, Saudi Arabia; ^3^Department of Medical Surgical College of Nursing, King Saud University, Riyadh, Saudi Arabia; ^4^Department of Nursing Administration and Education, College of Nursing, King Saud University, Riyadh, Saudi Arabia; ^5^Department of Optometry, College of Applied Medical Sciences, King Saud University, Riyadh, Saudi Arabia

**Keywords:** thyroid disease, hypothyroid, signs, genetics, smoking, healthcare

## Abstract

**Background and objective:**

In the healthcare context, healthcare personnel are available to help patients according to their requirements. However, having sufficient knowledge of many elements of diseases before graduation may have a good impact on clinical practices later in one's career. As a result, the purpose of this study was to assess the clinical knowledge and perceptions of healthcare students in Saudi Arabia about thyroid cancer (TC).

**Methods:**

A cross-sectional study was conducted in King Saud University from August 2021 to November 2021, using a validated self-reporting online survey. The data collection was carried out among senior healthcare students, including pharmacy, nursing, and medical students of both genders, who were Arabic speakers. The data were analyzed using the Statistical Package for the Social Sciences version 26 for Windows (SPSS).

**Results:**

There were 141 healthcare students who responded, with 46.8% (*n* = 66) being pharmacy students, 28.4% (*n* = 40) being nursing students, and 24.8% (*n* = 35) being medical students. Male participants made up the majority of them (52.5%). Lump or swelling in the neck was described as the most common early indicator of TC by 54.6% (*n* = 77), followed by difficulty in swallowing by 34.04% (*n* = 48), and pain in the neck by 24.8% (*n* = 35). Female participants accounted for 44.7% (*n* = 63) of those with thyroid dysfunction, according to the study. About 55.3% of the participants (*n* = 78), reported that they had sufficient knowledge about TC. The knowledge score differed significantly by gender; female participants (60.3%) (*n* = 47) were more knowledgeable than male participants (30.7%) (*n* = 31) (*p* = 0.049).

**Conclusion:**

This study depicts that half of the healthcare students thatwere knowledgeable about TC had positive perceptions about the causes of diseases. Furthermore, we also recommend arranging awareness programs for the students by the university officials to overcome the knowledge gap.

## Introduction

Endocrine diseases, particularly thyroid diseases, have become more common over time. The thyroid gland is a small butterfly-shaped gland that sits beneath the neck and regulates metabolism, growth, and development in the human body by the continuous secretion of thyroid hormones (1, 2). Thyroid disease is more common during adolescence or during pregnancy, especially in women between the ages of 20 and 45, therefore its risk factors have gotten a lot more attention (3–5). According to recent estimates, undiagnosed subclinical hypothyroidism was 4.11% in Europe, while the prevalence of overt hypothyroidism was 0.65%, for a total prevalence of 4.70% (6). In the United States, the American Thyroid Association estimated that at least 20 million US adults have some form of thyroid disease; interestingly, the majority of the thyroids were unaware of their disease status (7). In Saudi Arabia, the overall prevalence was 49.76%, among which subclinical hypothyroidism was the most prevalent type (3922/9992), followed by primary hypothyroidism (530/9992) (8). However, studies found that the prevalence of thyroid dysfunction varies by age, gender, race/ethnicity, geographical distribution, and the amount of dietary iodine intake (8, 9).

Hypothyroidism is a type of thyroid disorder in which the thyroid gland does not generate adequate hormones. Hypothyroidism is more common in women than in men, according to the US Department of Health and Human Services; hyperthyroidism, on the other hand, happens when your thyroid overworks and produces more hormones than it should. Hyperthyroidism is more frequent in women than in hypothyroidism (10). On the other hand, a previous study among Americans reported the prevalence of TC mostly at a median age of 47 years in women and 53 years in men. (11). However, TC is the most frequent and widespread of all endocrine cancers. (12–14) Despite its prevalence, it only accounts for roughly 2% of all malignant tumors in the United States (15).

According to a study conducted by Alyahya and colleagues among citizens of Saudi Arabia's eastern province, half of the participants, 44.7% (394), had insufficient information regarding thyroid condition (16). Furthermore, previous data revealed that approximately half of the people in the study were unaware of thyroid symptoms and risk factors (17, 18). Thyroid abnormalities are more common in women than men in the Arabic population, with a peak age of more than 30 years, and are connected to an increased risk of anemia. (8). Another recent study by Iqbal et al. among university students reported knowledge deficiency with respect to many aspects of thyroid disease (17). Another recent study among medical students concluded insufficient knowledge and awareness of predisposing variables and TC screening (18).

Students, primarily pharmacists, nurses, and medical professionals, are on the 'front lines' in the healthcare setting, ready to assist patients as needed. Prior to graduation, having a sufficient understanding of many elements of diseases may have a good impact on clinical practices later in work. In Saudi Arabia, there is a scarcity of studies on TC's knowledge, risk factors, awareness, and prevention practices among senior healthcare students. A study of the literature revealed that no research on senior pharmacy, nursing, or medical students' understanding of various aspects of thyroid disease had been conducted in Saudi Arabia. Therefore, this study aimed to evaluate the healthcare students' clinical knowledge and perceptions of developing TC in Saudi Arabia.

## Methods

### Study design, settings, population

A cross-sectional study was conducted at King Saud University over 4 months, from August 2021 to November 2021. It is a self-reporting questionnaire-based study including both genders of senior healthcare students who were currently pursuing their graduation and were from the branches like pharmacy, nursing, and medical students, who were Arabic speakers. Prior to data collection, the study protocol was reviewed and approved by the Institutional Review Board (IRB) at King Saud University Medical City (KSUMC), Riyadh, Saudi Arabia. At the beginning of the study and following the questionnaire, a disclosing statement followed by complying and an agreement to use filled information for publication purposes were highlighted. Healthcare students who have recently joined the courses and students from other disciplines were excluded from the study.

The required sample size for this study was obtained using the Rao soft sample size calculator (http://www.raosoft.com/samplesize.html.) with a 95% confidence level and a pre-determined margin of error of 5% from approximately 200 senior residential students (pharmacy, nursing, and medicine) on the KSU campus. Because we were unsure of the potential results for each question, we assumed that the response distribution for each question would be 50%. We calculated a sample size of 132 students, but we decided to survey 200 students to ensure greater reliability.

### Study questionnaire, data collection, and source

The questionnaires were prepared after an extensive literature review using similar studies published elsewhere (16, 17). The designed questionnaire was validated in two steps. First, the initial draft was evaluated by a research expert in the related field; second, a pilot study was conducted among a randomly selected sample of 10 students to give their opinions. Amendments from the pilot study were made, and the final draft of the questionnaires was sent to the targeted participants. The reliability test was done by calculating Cronbach's alpha using SPSS v.26, and the value of 0.76 indicated questionnaires suitable to carry out the study. The data of the pilot study were not included in the final analysis.

The questionnaire was divided into 3 sections. Section one includes the participant's demographics, including age, gender, profession, presence of diseases, and specific clinical information with a total of 9-items. The second section contains questions about the knowledge of signs and symptoms of thyroid disease with a total of 10 items. The last part of the study asks about participants' perceptions regarding a person's chance of developing TC with a total of 10 items. The responses to the last section of the questionnaires were recorded on a 5-point Likert scale. A score of 5 indicates disagree strongly, 4 indicates disagree, 3 indicates neutral, 2 indicates agree, and 1 indicates strongly agree. The knowledge score of thyroid disease was calculated for each item by allocating 'one' to the correct answer and 'zero' to the wrong answer.

We chose a convenience sampling procedure to collect the data from the targeted population. Convenience sampling is a non-probability sampling technique in which study participants are chosen based on a set of metrics, like as accessibility at a specific time, willingness to participate, ease of access, and proximity to the investigators.

The data were collected primarily from senior students at KSU's College of Pharmacy, nursing and medicine, who were pursuing their degrees. The participants were approached for data collection by a researcher from the College of Pharmacy's Clinical Pharmacy Department. The purpose of the study was presented at the beginning of the study questionnaire, additionally, prior to approaching the questionnaire, there was a statement about the consent. The information was gathered by paying visits to the group leaders of each course, and reminders were sent to fill out the questionnaires.

### Statistical analysis

The data were analyzed using the Statistical Package for the Social Sciences (SPSS) version 26 for Windows (SPSS Inc., Chicago, Illinois). Descriptive statistics was used to summarize the demographic characteristics. All statistical tests (Chi-square/Fisher exact test) were performed at a significance level of α = 0.05 and a 95% confidence interval (CI) for statistically significant differences between the variables.

## Results

### Demographic information of the participants

**Figure 1:**
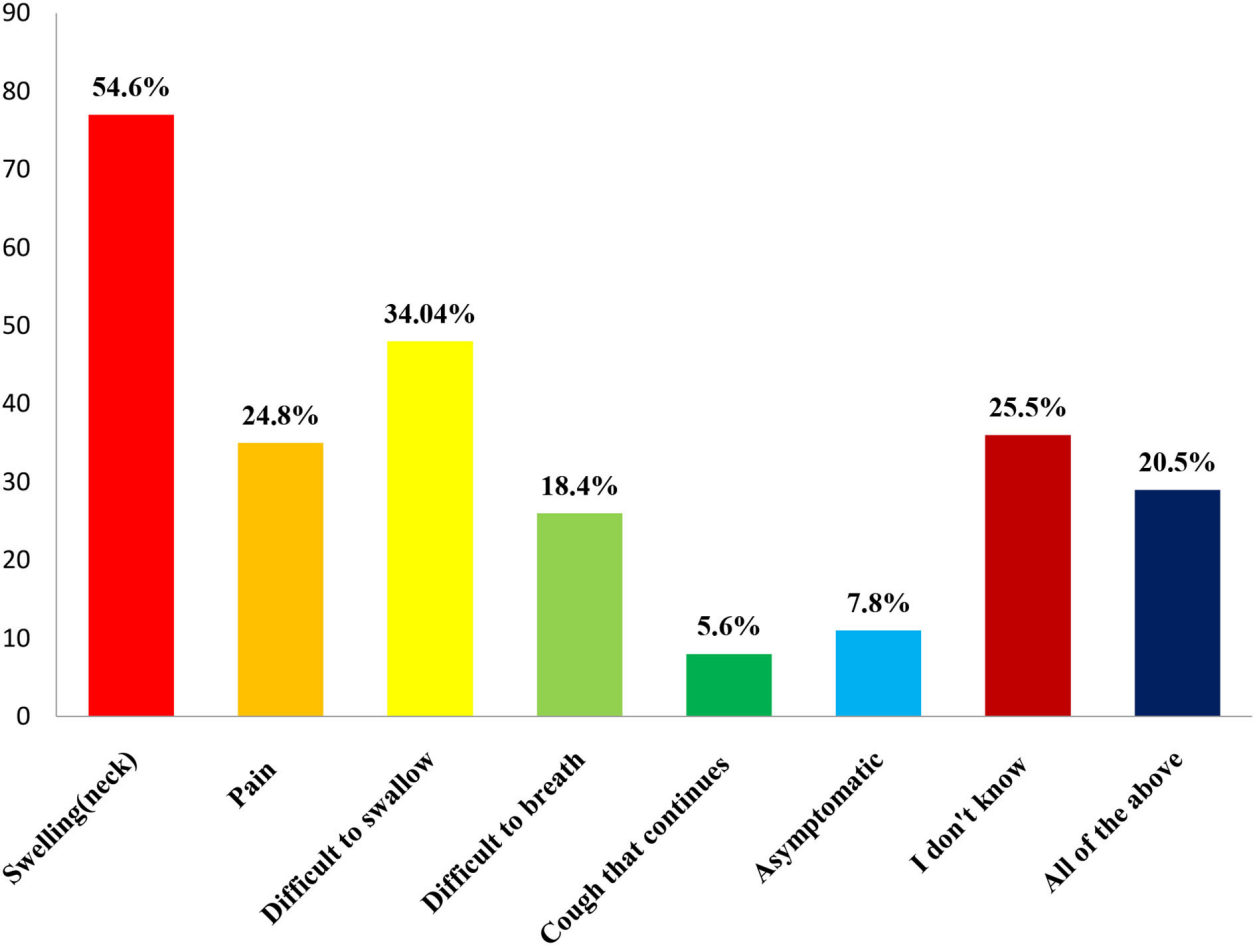
Knowledge regarding early signs of thyroid cancer.

In this study, a total of 200 healthcare students were approached, with 59 responses being discarded due to missing responses or incompleteness. The questionnaire was completed by 141 students, resulting in a response rate of 70.5%. The majority of the responders (46.8%, or *n* = 66) were pharmacists, while 28.4% (*n* = 40) were nurses, and 24.8% (*n* = 35) were medical students. Most of the students (52.5%) were male participants between the ages of 20 and 22, with the clear majority being Saudis (*n* = 108, 76.6%). However, a large proportion (90.8%) had free from chronic diseases when asked students the most common early signs of TC more than half of them cited lump or swelling in the neck 54.6% (*n* = 77), followed by difficulty in swallowing 34.04% (*n* = 48), pain in the neck, and sometimes in ears 24.8% (*n* = 35) (Figure1).

The most common diagnostic methods for screening of TC are neck palpation and thyroid ultra-sonography (manual examination and ultrasound technique) (40.42%, *n* = 57), followed by biopsy (38.29%, *n* = 54), and hormonal level testing (36.17%, *n* = 51). Women (44.7%, *n* = 63) are the most affected gender by thyroid dysfunction. Around 40.4% (*n* = 57) of the participants believed that iodine is required to synthesize thyroid hormones, and 45.4% of them believed that thyroid function impacts the menstrual cycle. The summary of the demographic information is presented in Table 1.

**Table 1:** Shows description of the study sample.

**Characteristics**	**Description**	**Frequency (*n*)**	**Percentage (%)**
Gender	Male Female	74 67	52.5 47.5
Age (years)	20–22 23–25 26–30	108 22 11	76.6 15.6 7.8
Nationality	Saudi Non-Saudi	137 4	97.2 2.8
Profession			
	Medical student Pharmacy student Nursing student	35 66 40	24.8 46.8 28.4
Presence of disease	Yes No	13 128	9.2 90.8
Diagnostic methods of thyroid cancer	Neck palpation/ ultrasonography Hormonal level testing Blood smear, Biopsy I Don't know	57 51 20 54 42	40.42 36.17 14.18 38.29 29.78
Most affected Gender	Male Female Both I Don't know	15 63 26 37	10.6 44.7 18.4 26.2
Thyroid function impact on the menstrual cycle	Yes No I Don't know	64 5 72	45.4 3.5 51.1
Iodine required for the synthesis of thyroid hormones	Yes No I Don't know	57 7 77	40.4 4.96 54.6

Unexplained lump or swelling was identified by more than two-thirds of the students as a warning sign of cancer, followed by persistent trouble swallowing (54%), unexplainable weight loss (52.5%), chronic unexplained discomfort (50.35%), and a persistent cough or hoarseness (43.97%). Table 2 lists other cancer warning indicators.

**Table 2:** Knowledge regarding warning signs for cancer.

**Characteristics**	**Description**	**Frequency (*n*)**	**Percentage (%)**
Unexplained lump or swelling could be a sign of cancer	Yes No I Don't know	92 25 24	65.24 17.73 17.02
Persistent unexplained pain could be a sign of cancer	Yes No I Don't know	71 32 38	50.35 22.69 26.95
Unexplained bleeding could be a sign of cancer	Yes No I Don't know	57 29 55	40.42 20.56 39.00
A persistent cough or hoarseness could be a sign of cancer	Yes No I Don't know	62 34 45	43.97 24.11 31.91
A persistent change in bowel or bladder habits could be a sign of cancer	Yes No I Don't know	41 31 68	29.07 21.98 48.22
Persistent difficulty swallowing could be a sign of cancer	Yes No I Don't know	77 19 45	54.60 13.47 31.91
A change in the appearance of a mole could be a sign of cancer	Yes No I Don't know	61 33 47	43.26 23.40 33.33
A sore that does not heal could be a sign of cancer	Yes No I Don't know	54 29 58	38.3 20.6 41.1
Unexplained weight loss could be a sign of cancer	Yes No I Don't know	74 23 44	52.5 16.3 31.2

The student's perception of a person's likelihood of having cancer is depicted in Table 3. In this study, the majority of the students believed that smoking can cause cancer (87.9%). Similarly, 83% of the students agreed that exposure to smoking can cause cancer. Likewise, more than two-thirds of the students agreed that eating red or processed meat and junk food once a day or more increases the risk of cancer, and more than half (56%) agreed that being overweight (BMI over 25) increases the risk of cancer, while 59.6% agreed that being over 70 years increases the risk of cancer. A total of 51.1% of the 141 students agreed that eating < five servings of fruits and vegetables per day increases the risk of acquiring cancer. Furthermore, 48.2% agreed that having a close relative with cancer is the chance of developing cancer. However, 54.6% of the students agreed that doing < 30 min of moderate physical activity five times a week could develop cancer.

**Table 3:** Perception regarding a person's chance of developing cancer.

**Variables**	**Mean ±Std**	**Strongly agree** ***n* (%)**	**Agree *n* (%)**	**Neutral** ***n* (%)**	**Strongly Disagree *n* (%)**	**Disagree** ***n* (%)**
Smoking any cigarettes at all	4.15 ± 1.53	97 (68.8)	27 (19.1)	12 (8.5)	2 (1.4)	3 (2.1)
Exposure to another person's cigarette smoke	3.36 ± 1.81	63 (44.7)	56 (38.3)	19 (13.5)	2 (1.4)	3 (2.1)
Eating less than five portions of fruit and vegetables a day	4.18 ± 1.98	17 (12.1)	30 (39.0)	45 (31.9)	19 (13.5)	5 (3.5)
Eating red or proceed meat and junk food once a day or more	3.95 ± 2.08	29 (20.6)	58 (41.1)	41 (29.1)	10 (7.1)	3 (2.1)
Being overweight (BMI over 25)	4.08 ± 2.00	26 (18.4)	53 (37.6)	46 (32.6)	11 (7.8)	5 (3.5)
Getting sun burnt more than once as a child	4.53 ± 1.99	30 (21.3)	28 (28.4)	48 (34.0)	20 (14.2)	3 (2.1)
Being over 70-year old	4.06 ± 2.09	29 (20.6)	55 (39.0)	43 (30.5)	12 (8.5)	2 (1.4)
Having a close relative with cancer	3.97 ± 1.52	43 (30.5)	25 (17.7)	37 (26.2)	19 (13.5)	17 (21.1)
Infection with HPV (Human Papilloma virus)	5.63 ± 2.01	31 (22.0)	25 (17.7)	61 (43.3)	16 (11.3)	8 (5.7)
Doing < 30 min of moderate physical activity five times a week	4.18 ± 2.03	23 (16.3)	54 (38.3)	40 (28.4)	18 (12.8)	6 (4.3)

In this study, 55.3% (*n* = 78) of the participants reported good knowledge of TC. The knowledge score is significantly different with respect to gender; 60.3% of the female participants (*n* = 47) and 39.7% of the male participants (*n* = 31) (*p* = 0.049) were knowledgeable. Although the knowledge score on the thyroid is not significantly associated with the student's age, the professional class had previous knowledge of thyroid disease and the presence of chronic disease (*p* > 0.005). The association between the knowledge score and participants' demographics is given in Table 4.

**Table 4:** Association between the participants' knowledge score levels and demographics.

	**Number of Respondents**	**Not Knowledgeable** **(*n* = 62; 44%)**	**Knowledgeable** **(*n* = 78; 55.3%)**	***p-* value**
**Age**				
20–22 years	Respondents	51	57	
	% within age	47.2%	52.8%	
23–25 years	Respondents	05	16	0.110
	% within age	23.8%	76.2%	
26–30 years	Respondents	06	05	
	% within age	54.5	45.5	
**Gender**				
Male	Respondents	35	31	0.049
	% within gender	53.0%	47.0%	
Female	Respondents	27	47	
	% within gender	36.5%	63.5%	
**Profession**				
Pharmacy	Respondents	29	36	
	% within professional class	44.6%	55.4%	
Nursing	Respondents	18	22	0.980
	% within professional class	45.0%	55.0%	
Medical	Respondents	15	20	
	% within professional class	42.9	57.1	
**Presence of disease**				
Yes	Respondents	06	07	
	% within presence of disease	46.2%	53.8%	
No	Respondents	56	71	0.887
	% within presence of disease	44.1%	55.9%	
**Heard about thyroid**				
Yes	Respondents	57	63	
	% within heard about thyroid	47.5%	52.5%	0.061
No	Respondents	05	15	
	% within heard about thyroid	25.0%	75.0%	

## Discussion

One of the most prevalent forms of disease these days is endocrine-related and most commonly known as TC, a cancerous tumor that is more common among people with a risk factor. However, in most cases, the specific cause is unknown. Globally, the rates of TC have risen dramatically. This cross-sectional study summarizes the knowledge and perceptions of TC among healthcare students in Saudi Arabia. In this context, the present study offers insight into the existing state of awareness among the healthcare students of King Saud University, Riyadh, Saudi Arabia. This study shows that more than half of the healthcare students have a good knowledge level of TC. These results were higher than a similar study by Iqbal et al. that revealed that almost all participants had poor knowledge about early signs, predisposing factors, and preventive practices of TC (17). On the contrary, a similar study conducted in Riyadh showed a similar result to our study, and it showed that the participants had a good level of knowledge (18). Another study conducted among women in India showed that most participants had inadequate knowledge about thyroid disorders (19). Our study reported that the knowledge score is significantly different concerning gender, 60.3% of the females (*n* = 47) and 39.7% of the males (*n* = 31) (*p* = 0.049) were knowledgeable. Another similar study was found to be having a knowledge ratio of 1:2.2 male to female (20).

The early sign of TC among this participant provides an exciting result. For instance, the current study population answered that 77% of the participants knew that lumps or swelling in the neck were early signs of TC. In contrast, a study in Saudi Arabia concluded that 70.6% of neck lumps could be a sign of TC (16). In this survey, more than half of the healthcare students agreed that persistent difficulty swallowing, unexplained weight loss, and persistent unexplained pain as the warning sign of cancer. Conversely, a previous study by Iqbal A et al. reported unexplained bleeding (45.9%), persistent difficulty in swallowing (42.5%), and unexplained weight loss (41.3%) as the early sign of TC (17).

Consistent with the present findings, it has been reported that exposure to smoking is the associated factor in the development of TC. On the other hand, a study conducted among residents of the Eastern Province, Saudi Arabia, showed that 40.5% agreed that smoking is a risk factor for thyroid diseases (16). Another recent study conducted among university students showed that 55.4% agreed that smoking causes the chances to develop TC (17, 21). One-third of the students believed that having a close relative with cancer is the cause of TC. A study conducted in Saudi Arabia among females showed that 68.5% of the participants reported that TC is often genetic (22).

Furthermore, the American Cancer Society and EndocrineWeb reported that family history, radiation exposure, sex, and age were the vital factors that can cause TC (23, 24). Increased public awareness about early signs of TC can improve overall disease diagnosis and treatment, morbidity, and death rates.

Iodine plays a crucial role in synthesizing thyroid hormones in the thyroid gland. At the same time, most of the participants in our study did not think that iodine was crucial for thyroid hormone synthesis. Nevertheless, many studies have shown that iodine deficiency is a risk factor for many thyroid gland diseases (25). This is mainly due to the lack of awareness of the disease among healthcare students. The majority of the students in our study opined that females are the most affected gender. Previous research on TC has shown that females are affected more frequently than males, consistent with our findings (17, 26, 27). The study participants in our study remained neutral on the statement that thyroid function impact the menstrual cycle; in contrast, university students in Pakistan (47.8%) reported that thyroid function impacts the menstrual cycle (17). This can be attributed to the efforts for creating awareness on TC among the different levels of healthcare students.

In this current research, we emphasize the importance of further studies that can evaluate the perceptions of healthcare students. This study provides a good platform for others to conduct research within the domains. However, the current study has some limitations. First, the results were based on a self-completed questionnaire, which may have increased the possibility of biases such as social desirability bias or recall bias. Second, the results were derived from a single institute in Saudi Arabia, thus making them not representative of others and not generalizable globally. Third, the study did not involve junior students as it was conducted among senior healthcare students of the university, given the more accessible access to students found while spreading the questionnaire. Despite these limitations, our study lays more emphasis on increasing the awareness of health in college students, especially toward the knowledge of thyroid and its complications to make them more competent in raising public health.

## Conclusion

This study depicts that half of the healthcare students who were knowledgeable about TC had positive perceptions of TC. The knowledge with respect to predisposing factors and early signs was inadequate and appropriate steps should be implemented to increase awareness to prevent the incidence of thyroid cancer. Thus, health education programs might help the participants understand and prevent the complications of TC and good adherence to treatment.

## Data availability statement

The original contributions presented in the study are included in the article/supplementary material, further inquiries can be directed to the corresponding author.

## Ethics statement

The studies involving human participants were reviewed and approved by King Saud University College of Medicine E-21-6371. The patients/participants provided their written informed consent to participate in this study.

## Author contributions

WS, OS, AA, MH, and MA-R conceived of this study and its design, conducted the data collection, reviewed and edited, performed the screening process, performed the content analysis and coding, and involved in interpreting the results. All authors have read and agreed to the published version of the manuscript.

## Funding

The author(s) disclosed receipt of the following financial support for the research, authorship, and/or publication of this article. This study was supported by the Research Supporting Project, King Saud University, Saudi Arabia, (RSP-2021/378) who provided funding for this work.

## Conflict of interest

The authors declare that the research was conducted in the absence of any commercial or financial relationships that could be construed as a potential conflict of interest.

## Publisher's note

All claims expressed in this article are solely those of the authors and do not necessarily represent those of their affiliated organizations, or those of the publisher, the editors and the reviewers. Any product that may be evaluated in this article, or claim that may be made by its manufacturer, is not guaranteed or endorsed by the publisher.
